# Rapid Detection of Carbapenemase-Producing Multidrug-Resistant (MDR) Pathogens by Modified Carba NP Test in Ventilator-Associated Pneumonia (VAP) in Elderly Patients

**DOI:** 10.7759/cureus.43895

**Published:** 2023-08-22

**Authors:** Rahil Pasha S A, Ruby Suresh Kumar Yadav, Md Iqbal Ahmed, Pratibha Chandra

**Affiliations:** 1 Department of Microbiology, Sri Devraj Urs Medical College, Kolar, IND; 2 Department of Microbiology, Employees' State Insurance Corporation (ESIC) Model Hospital, Gurugram, IND; 3 Department of Microbiology, Employees' State Insurance Corporation (ESIC) Medical College, Gulbarga, IND; 4 Department of Microbiology, Employees' State Insurance Corporation (ESIC) Medical College, Patna, IND

**Keywords:** vap, metallo beta-lactamase, carbapenemase, carbanp test, geriatric vap

## Abstract

Background

Ventilator-associated pneumonia (VAP) is defined as pneumonia that develops 48 hours or more after endotracheal intubation or tracheostomy and is brought on by infectious organisms that are not present or incubating during mechanical ventilation. Multidrug-resistant organisms originate primarily from the hospital environment and significantly contribute to ventilator-associated pneumonia. These organisms pose a severe threat, leading to a higher mortality rate due to their resistance to more potent antibiotics.

Methods

The study aims to assess the efficacy of the modified Carba NP test in detecting carbapenemase-producing bacteria in geriatric VAP patients.

Results

Forty (38 gram-negative and 2 gram-positive) pathogens were isolated from VAP patients. The isolates were identified using standard laboratory protocol; *Acinetobacter spp*. (n=16; 40% ), followed by *Klebsiella pneumoniae *(n=13; 32.5%), is the most common organism isolated. Seventeen (44.73%) were multi-drug resistant gram-negative bacteria. The carbapenemase producers were detected by the Kirby-Bauer disc diffusion method and compared with the modified Carba NP test with a turnaround time of 12-18 hrs in comparison to the disk diffusion test which requires additional 12hrs. Carbapenemase production was seen in 12 (70.59%) MDR isolates (7-*Acinetobacter spp*, 3-*Klebsiella pneumonia*, 1-*Escherichia coli,* and 1-*Pseudomonas aeruginosa)*.

Conclusion

Modified Carba NP can be used as a rapid test to detect carbapenemase production, and it can replace the traditional disk diffusion method of detecting carbapenemase production. This test plays a crucial role in the management of critical patients by saving 12-18 hours to determine the most appropriate and effective antibiotic treatment. This timely decision is essential in preventing sepsis caused by localized infections.

## Introduction

Multi-drug resistance in gram-negative bacteria (GNB) is a worldwide concern, posing a significant threat to public health and healthcare systems worldwide. Carbapenems are the last resort for treating infections caused by multidrug-resistant bacteria [1]. “The extensive utilization of carbapenems in clinical settings has resulted in the emergence of antibiotic resistance against these drugs. Carbapenem resistance can result from Carbapenemase production, reduced outer membrane permeability with overexpressed AmpC/ESBL, or overexpression of efflux pumps” [1, 2]. Ventilator-associated pneumonia is a feared hospital-acquired infection, posing significant risks for geriatric patients due to physical impairments, age-related immunological changes, and chronic cognitive alterations, reducing host resistance [3].

According to the Global Burden of Disease (GBD-2015) report, chronic obstructive pulmonary disease (COPD) and lower respiratory tract infections (LRTI) rank as the third and fourth leading causes of death in geriatric patients[4]. VAP is associated with prolonged mechanical ventilation and intensive care unit (ICU) stays [5]. The impact of infectious diseases on geriatric patients should be assessed beyond mortality rate, morbidity, and quality of life [6]. The gram-negative bacterium was considered multi-drug resistant (MDR) when resistant to a representative drug from these three groups of antibiotics, β-lactam, aminoglycoside, and quinolone [6]. The expansion of new resistance mechanisms in drug-resistant pathogens leads to an increase in antimicrobial resistance; especially It is a global health threat in the future if bacteria that are multi- and pan-resistant (also known as "superbugs") produce infections that cannot be treated with existing antimicrobial medicine, such as antibiotics [7]. As drug-resistant bacteria increase, hospital infection control practices become vital in halting their progression and transmission [8].

Both phenotypic and molecular methods are used to identify carbapenemase production, with molecular techniques considered the gold standard for detection [9]. While molecular methods provide confirmation, their immediate obtainability and the number of targets detected may be limiting factors in testing [10]. A novel, cost-effective, modified Carba NP test for carbapenemase detection based on the principle of acidimetry has been developed by Rudresh et al. [11]. The acidimetric method involves beta-lactam ring hydrolysis, leading to a pH decrease and a color change of the phenol red indicator from red to yellow [11]. The present study aimed to evaluate the clinical utilization of the modified Carba NP test for detecting carbapenemase-producing bacteria.

## Materials and methods

Study design

An unblinded prospective study was initiated following approval from the institutional ethics committee.

Source of data

Endo tracheal (ET) aspirate and broncho-alveolar lavage (BAL) from geriatric patients submitted to the diagnostic microbiology laboratory to a 750 bedded tertiary care hospital.

Study subjects

Inclusion Criteria

The inclusion criteria were defined as elderly patients aged 60 years or above diagnosed with BAL & ET aspirate.

Exclusion Criteria

The exclusion criteria included open cases of pulmonary tuberculosis, patients on chronic suppressive antimicrobial therapy and retro-positive patients.

Patients provided informed consent, and strict confidentiality was ensured throughout the study.

Sample collection

Sample Method

Simple random sampling was identified as the most suitable sampling method for this study.

Collection of ET aspirate

ET aspirate was collected using 12 French (Fr) tracheal aspiration probes. The probe was introduced through the ET tube until resistance (level of the carina in the trachea) was encountered. It was retracted by 2cm to release the vacuum. The probe was carefully removed using turning movements, and secretions were aspirated into a sterile collector tube [12, 13].

Collection of BAL

An intrabronchial injection of saline (100-300 mL) was used to obtain interstitial and alveolar proteins from the lungs. Aspirate consisting of deep desquamated host cells and secretions was collected [12, 13].

Identification of bacterial isolates

Processing of Samples

Tracheal aspirate/ BAL: Utmost purulent portion of tracheal secretion was taken; a 0.1 ml sample was diluted in a 9.9 ml sterile physiological solution, and 0.01 ml was seeded (calibrated loop) on MacConkey agar, blood agar & chocolate agar and incubation at 35 ± 1ºC for 24 to 48 hours (chocolate agar, in canophilia (5% of CO2) at 35 ± 1ºC for 24 to 48h) [14].

Plates were assessed for growth at 24 and 48 hours. Bacterial isolates grown in culture were recognized using Gram staining and biochemical reactions by standard microbiological techniques. Each colony corresponded to 20,000CFU/ml and was considered positive when the count was ≥105CFU/ml [14].

Modified Carba NP (mCNP) test

**Figure 1:**
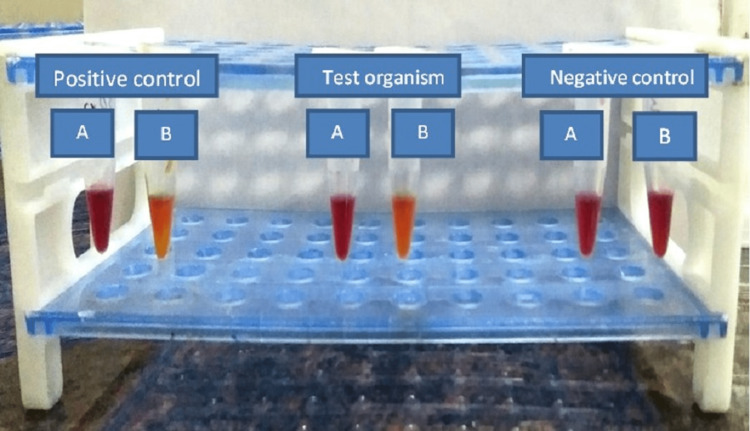
Modified Carba NP (mCNP) test

A modified Carba NP (mCNP) test was performed from the direct plate, and the results were analyzed. CNP solution-A was prepared by the addition of phenol red (0.05%) and ZnSO4.7H2O (0.1 mmol/L) to clinical laboratory chemical water; pH was adjusted to 7.8 ± 0.1, and the solution was stored at 4°C in amber-colored bottles for up to 15 days. The solution-B was freshly prepared by addition of 12 mg/ml imipenem‑ cilastatin injectable form (doubling the amount to compensate for the cilastatin component; equivalent to 6 mg/ml of imipenem standard grade powder) to A solution and stored at 40C till use. 10μl of a bacterial colony from 18 to 24 h growth culture from sheep blood agar were re-suspended in 200μl of 5 M NaCl solution and vortexed for five seconds. A 100μl of inoculum was added to two microcentrifuge tubes labeled "a" and "b." Reagents A and B were added to tubes A and B, incubated at 37°C, and read at two hours. The test was considered positive when tube "a" was red and tube "b" was orange/yellow. In a negative test, both tubes remained red” [11] (Figure 1).

## Results

Forty organisms were isolated, among which 38 were gram-negative and two were gram-positive isolates. Among 38 Gram-negative bacteria, 17 (44.73%) were MDRs. 56.25% of *Acinetobacter* spp. were MDRs. Overall, MDR prevalence was 44.73% (Table 1).

**Table 1:** Distribution of multidrug-resistant (MDR_ gram-negative bacteria among endotracheal aspirate & broncho-alveolar lavage (n=38)

Bacterial isolates	MDR	Percentage (%)
Acinetobacter spp. (n=16)	9	56.25%
Klebsiella pneumoniae (n=13)	5	38.46%
Pseudomonas aeruginosa (n=7)	2	28.57%
Escherichia coli (n=2)	1	50.00%
Total (n=38)	17	44.73%

Males (53%) were predominantly infected; the 60-79 years age group was most commonly involved (Table 2).

**Table 2:** Distribution of multidrug-resistant (MDR) gram-negative bacteria among tracheal aspirate & broncho-alveolar lavage (n=17) concerning age

Age	Female	Male	Total
60-79 years	5 (29.4%)	7 (41.2%)	12 (70.6%)
≥80 years	3 (17.7%)	2 (11.8%)	05 (29.4%)
Total	8 (47.0%)	9 (53%)	17 (100%)

Among the MDR isolates, cefoperazone-sulbactam (41.18) unveiled the highest antibiotic sensitivity, followed by amikacin, meropenem, and piperacillin-tazobactam (29.42). Conversely, among non-MDR isolates, amikacin (76.2%) displayed the highest sensitivity to antibiotics, followed by cefoperazone-sulbactam and gentamicin (both at 71.43%) (Table 3).

**Table 3:** Antibiotic resistance comparison among multidrug-resistant (MDR) and Non-MDR gram-negative isolates (n=38).

Antibiotic	MDR (n=17)	% of Resistance	Non-MDR (n=21)	% of Resistance
Ciprofloxacin	15	88.23	10	47.62
Ceftazidime	15	88.23	13	61.90
Gentamycin	15	88.23	6	28.57
Cefoperazone	14	82.35	15	71.43
Aztreonam	14	82.35	13	61.90
Imipenem	14	82.35	8	38.09
Piperacillin	13	76.47	14	66.67
Piperacillin-tazobactam	12	70.58	8	38.09
Meropenem	12	70.58	8	38.09
Amikacin	12	70.58	5	23.81
Cefoperazone-sulbactam	10	58.82	6	28.57

Carbapenemase production (70.59%) was the primary resistance mechanism among MDR isolates, with *Acinetobacter* spp accounting for seven cases, *Klebsiella pneumoniae *for three cases, and *Pseudomonas aeruginosa* and *Escherichia coli* each contributing one case. The most common mechanism was AmpC (11.8%), with *Klebsiella pneumoniae* and *Escherichia coli* each having one case. Additionally, *Klebsiella pneumoniae* showed ESBL production in 5.9% of cases. Metallo-beta-lactamase production was observed in 23.53% of isolates among carbapenemase producers (Table 4).

**Table 4:** Beta-lactamase production in multidrug-resistant (MDR) gram-negative isolates (n=17)

Mechanism of resistance		Frequency	Percentage (%)
Carbapenemase	Metallo-βlactamase (n=5)	12	70.59
	Non-Metallo- βlactamase (n=7)		
AmpC		2	11.8
ESBL		1	5.9
ESBL+AmpC		1	5.9

All carbapenemase-producing isolates identified through the disk diffusion method were confirmed as positive by the modified Carba NP test (Table 5).

**Table 5:** Comparison of the Carba NP test (rapid detection) with carbapenemase production using the disk diffusion method (n=12)

Method of detection	Frequency
Carbapenemase detection by disk diffusion	12
Carba NP Positive isolates test	12

Diabetes is the most common risk factor, followed by smoking and previous history of COPD (Table 6).

**Table 6:** Risk factors associated with multidrug-resistant (MDR) Positive VAP infections (n=17)

Risk factor	Quantity	Percentage (%)
Diabetic	9	52.9
Smoking	8	47.1
Previous COPD	7	41.2
Poor oral hygiene	5	29.4
Alcohol	5	29.4
Cardiac diseases	3	17.65
CVA/Hemiparesis	2	11.76
Carcinoma lung	1	5.88
Kidney disease	1	5.88

Radiological correlation (n=17)

All patients had their chest x-rays correlated. Six (35.3%) B/L interstitial or alveolar infiltration was seen in the individuals, five (29.4%) patients had pneumonic changes (consolidation), and one (5.9%) patient had consolidation with carcinoma (CA) lung.

## Discussion

The modified Carba NP test is a colorimetric assay for detecting carbapenem hydrolysis, serving as a confirmation test for carbapenemase production in Enterobacteriaceae, Acinetobacter spp., and P. aeruginosa” [15].

The most common and potentially fatal sepsis experienced by hospitalized patients is ventilator-associated pneumonia (VAP). This condition can lead to higher mortality and morbidity rates, especially as multidrug-resistant organisms (MDRO) become more prevalent [16]. Therefore, it becomes essential to carefully evaluate the severity of clinical symptoms and the risk of acquiring MDRO infections to establish a precise and efficient diagnostic approach. Analyzing the risk factors associated with MDRO-induced VAP proves to be a beneficial strategy.

Carbapenem resistance in gram-negative bacteria is critical, especially for VAP patients in critical care units [17]. In our study, we found that *Acinetobacter* and *Klebsiella* were the most predominant carbapenemase-producing organisms (Table 1), which is in concordance with Nordmann and Poirel's research, where *P. aeruginosa* and *A. baumannii* were the most common microorganisms associated with carbapenem resistance [1]. Early diagnosis of carbapenemase-producing bacteria is vital for selecting the proper antimicrobial treatment, improving outcomes, and implementing effective infection control measures [18].

Forty organisms were isolated from microbiologically confirmed VAP patients. In our study, 53% of the patients were male, showing a higher prevalence of infections by Multi-drug resistant organisms (Table 2). This aligns with research by Ahmed W et al. and Farag et al. in south India, where pneumoniae prevalence in the elderly was 72.91%, with males comprising 60.42% and *Acinetobacter baumanii* being the most common bacteria causing VAP (36.11%) [17, 19].

The higher incidence can be linked to prolonged hospital stays of VAP patients, increasing their vulnerability to nosocomial infections and invasive procedures, often leading to improper use of broad-spectrum antibiotics as initial treatment. Moreover, the prevalence of smoking among South Indian males can impair the normal ciliary function of the respiratory tract, making them more susceptible to infections.

Among 40 strains isolated from VAP patients, 17 isolates were multidrug-resistant (Table 2). The test microorganisms were resistant to widely used therapeutic antibiotics like ciprofloxacin, ceftazidime, gentamycin, cefoperazone, imipenem, and meropenem. Cefoperazone-sulbactam (sensitivity rate 41.18%), followed by Amikacin (sensitivity rate 29.42%), was our study's most effective antibiotic for multidrug-resistant organisms (Table 3). Chen et al. found that cefoperazone-sulbactam is a suitable antibiotic treatment for HAP/VAP caused by most GNBs [20]. Considering amikacin's renal toxicity, cefoperazone-sulbactam would be the recommended empirical treatment for suspected VAP. Avibactam is a novel beta-lactamase inhibitor that inhibits the serine carbapenemase enzyme (ambler A) but doesn't have metallase (ambler B) action"[21].

The analysis of beta-lactamase production in MDR gram-negative isolates revealed that carbapenemase, extended-spectrum-beta-lactamases (ESBL's), and antimicrobial resistance enzymes (AmpC) production are the primary mechanism of drug resistance in these strains (Table 4). “A study by Gao et al. on molecular detection and genetic characteristics of carbapenem-resistant Enterobacterales (CRE) observed that the gene of carbapenemase and ESBL's were the most important factors involved in mechanisms of drug resistance” [22].

According to a study by Nunez et al., major risk factors for developing VAP include COPD, congestive heart failure, diabetes, malignant diseases, chronic renal failure, obesity, and immunosuppression which were concordant with our study [23].

The infection control specialist and clinicians can take the right action immediately by using the modified Carba NP test to detect carbapenemase-producing bacteria responsible for causing hospital-acquired infections.

## Conclusions

Modified Carba NP test can be used in the laboratory to detect carbapenemase resistance and is essential in critical patients as carbapenems are used in empirical antibiotic treatment. This test significantly reduces the decision time for definitive antibiotic treatment in critical patients by saving 12-18 hours. High antibiotic resistance was observed in the study, and *Acinetobacter spp*. was the primary causative agent of ventilator-associated pneumonia (VAP).

It is possible to stop the spread of multidrug-resistant pathogens in hospital settings by early detecting carbapenemase-producing bacteria using the modified Carba NP test which is a quick, reliable, and affordable option that can be used regularly in nations with limited resources.

## References

[1] Nordmann P, Poirel L, Dortet L (2012). Rapid detection of carbapenemase-producing enterobacteriaceae. Emerg Infect Dis.

[2] Tijet N, Boyd D, Patel SN, Mulvey MR, Melano RG (2013). Evaluation of the carba NP test for rapid detection of carbapenemase-producing enterobacteriaceae and pseudomonas aeruginosa. Antimicrob Agents Chemother.

[3] van de Nadort C, Smeets HM, Bont J, Zuithoff NP, Hak E, Verheij TJ (2009). Prognosis of primary care patients aged 80 years and older with lower respiratory tract infection. Br J Gen Pract.

[4] GBD 2015 Mortality and Causes of Death Collaborators (2016). Global, regional, and national life expectancy, all-cause mortality, and cause of death, 1980 2015: a systematic analysis for the global burden of disease study 2015. Lancet.

[5] Papazian L, Klompas M, Luyt CE (2020). Ventilator-associated pneumonia in adults: a narrative review. Intensive Care Med.

[6] Gavazzi G, Herrmann F, Krause KH (2004). Aging and infectious diseases in the developing world. Clin Infect Dis.

[7] Pasha SAR, AbdulNaseer A, Hajira SN (2020). Microbiological profile of ventilator associated pneumonia (VAP) in geriatric patients and their antibiotic susceptibility pattern with detection of MRSA, ESBLs and MBLs in intensive care unit of a tertiary care center from South India. Int J Curr Microbiol App Sci.

[8] Pasha SAR, Bhat P, AbdulNaseer A (2020). Ventilator associated pneumonia (VAP) with multidrug-resistant (MDR) pathogens in geriatric patients; risk factors and their antibiotic susceptibility pattern with detection of MRSA, ESBLs and MBLs in intensive care unit. Int J Sci Res.

[9] Österblad M, Hakanen AJ, Jalava J (2014). Evaluation of the carba NP test for carbapenemase detection. Antimicrob Agents Chemother.

[10] Vasoo S, Cunningham SA, Kohner PC (2013). Comparison of a novel, rapid chromogenic biochemical assay, the carba NP test, with the modified Hodge test for detection of carbapenemase-producing gram-negative bacilli. J Clin Microbiol.

[11] Rudresh SM, Ravi GS, Sunitha L, Hajira SN, Kalaiarasan E, Harish BN (2017). Simple, rapid, and cost-effective modified Carba NP test for carbapenemase detection among gram-negative bacteria. J Lab Physicians.

[12] Frota OP, Ferreira AM, Barcelos Lda S, Watanabe E, Carvalho NC, Rigotti MA (2014). Collection of tracheal aspirate: safety and microbiological concordance between two techniques (Article in Portuguese). Rev Esc Enferm USP.

[13] Koenig SM, Truwit JD (2006). Ventilator-associated pneumonia: diagnosis, treatment, and prevention. Clin Microbiol Rev.

[14] J G Collee, W Marr (2014). Specimen collection, culture containers, and media. Mackie and McCartney Practical Medical Microbiology.

[15] Clinical and Laboratory Standards Institute (2015). Performance standards for antimicrobial susceptibility testing; 25th informational supplement. https://www.scirp.org/(S(lz5mqp453edsnp55rrgjct55))/reference/ReferencesPapers.aspx?ReferenceID=1954720.

[16] Jean SS, Chang YC, Lin WC, Lee WS, Hsueh PR, Hsu CW (2020). Epidemiology, treatment, and prevention of nosocomial bacterial pneumonia. J Clin Med.

[17] Farag AM, Tawfick MM, Abozeed MY, Shaban EA, Abo-Shadi MA (2020). Microbiological profile of ventilator-associated pneumonia among intensive care unit patients in tertiary Egyptian hospitals. J Infect Dev Ctries.

[18] Ohsaki Y, Kubo R, Hobson J (2018). MASTDISCS combi carba plus, a simple method for discriminating carbapenemase-producing enterobacteriaceae, including OXA-48-type producers. Microbiol Immunol.

[19] Ahmed W, Rana M N, Muzaffar N A, Abbassi S (2014). Microorganisms related with ventilator associated pneumonia (VAP) and their antibiotic sensitivity pattern. Journal of Rawalpindi Medical College.

[20] Chen CH, Tu CY, Chen WC (2021). Clinical efficacy of cefoperazone-sulbactam versus piperacillin-tazobactam in the treatment of hospital-acquired pneumonia and ventilator-associated pneumonia. Infect Drug Resist.

[21] van Duin D, Lok JJ, Earley M (2018). Colistin versus ceftazidime-avibactam in the treatment of infections due to carbapenem-resistant enterobacteriaceae. Clin Infect Dis.

[22] Gao B, Li X, Yang F, Chen W, Zhao Y, Bai G, Zhang Z (2019). Molecular epidemiology and risk factors of ventilator-associated pneumonia infection caused by carbapenem-resistant enterobacteriaceae. Front Pharmacol.

[23] Núñez SA, Roveda G, Zárate MS, Emmerich M, Verón MT (2021). Ventilator-associated pneumonia in patients on prolonged mechanical ventilation: description, risk factors for mortality, and performance of the SOFA score. J Bras Pneumol.

